# ‘You don’t need schooling—just take the pills and don’t stop.’: Pathways between formal education and chronic HIV care in Malawi

**DOI:** 10.1016/j.ssmqr.2025.100599

**Published:** 2025-07-17

**Authors:** Stephanie Chamberlin, Misheck Mphande, Pericles Kalande, Khumbo Phiri, Kathryn Dovel

**Affiliations:** a Department of Population Health Sciences, University of Wisconsin-Madison School of Medicine and Public Health, USA; b Partners in Hope Medical Center, Lilongwe, Malawi; c Department of Medicine and Division of Infectious Diseases, David Geffen School of Medicine at University of California Los Angeles, Los Angeles, CA, USA

**Keywords:** HIV, Treatment adherence, sub-Saharan Africa, Qualitative, Chronic care, International education, Formal schooling

## Abstract

Research on chronic care management from other settings indicates that people’s formal education (e.g., primary, secondary, and tertiary schooling) may confer important cognitive skills and material resources to help them manage their chronic HIV care and treatment. However, recent population-level findings from southern and eastern Africa suggest no statistical relationship between education and chronic HIV care. To gain additional insight into these puzzling findings, we draw on in-depth interviews with HIV care clients in Malawi to understand how people acquire education-related resources and how they use such resources to support their HIV care. These interviews suggest that education-related cognitive and material resources are central to HIV care management, but such resources are not necessarily gained through formal schooling. Importantly, HIV clients use a variety of strategies to overcome their limited skills and resources, often relying on the literacy and material resources of their family and community to facilitate their HIV care. Taken together, these findings provide new insight into the mechanisms that link or attenuate education-health relationships in different contexts globally. Further, this work informs the development of more equitable chronic care interventions for meeting the needs of people across different education levels.

## 1. Introduction

Governments and international organizations working in sub-Saharan Africa have long heralded formal schooling as a key social policy lever for improving population health. Such assertions are largely based on well-documented positive associations between women’s education and maternal and child health in the region (LeVine et al., 2011; Mensch et al., 2019). However, the association between education and chronic care have received less attention—a concerning gap amidst rising rates of chronic health conditions across sub-Saharan Africa. To gain insight into this gap, we look at the case of HIV, which is one of the most commonly treated chronic conditions in the region (Vorkoper et al., 2018).

Existing evidence suggests that formal educational attainment is largely unrelated to chronic HIV care management in lower resource settings. A recent study across seven southern and eastern African countries shows no population-level associations between formal education and effective chronic HIV care management—specifically HIV treatment adherence measured via viral load suppression (Chamberlin, 2022). These findings held in sensitivity analyses using a continuous measure of ‘years of education’ to capture the benefits of each additional year of schooling, as well as tests with categorical cut-offs for levels of education—such as primary, secondary, and tertiary; or no versus any schooling. Across sub-Saharan Africa, similar null associations are frequently reported between HIV treatment adherence and formal education (Heestermans et al., 2016). These patterns mirror null findings for diabetes self-management in relation to educational attainment in six low and middle income countries—the only other study we are aware of that directly examines formal education-chronic care management relationships at the population level in similar settings (Lamb et al., 2021).

These findings are perplexing, given that the skills and resources needed to effectively manage chronic care are theoretically facilitated by an individual’s formal education. Common chronic care tasks—for HIV and other chronic conditions alike—include routine health care appointments, timely prescription refills, and on-going adherence to treatment. These tasks require clients to exhibit consistent motivation, executive functioning skills, and resources (e.g., knowledge, income, and literacy), all of which are often empirically linked to greater educational attainment (Mirowsky, 2017). This introduces important questions about *why* education does *not* positively influence chronic HIV care management in the context of eastern and southern Africa.

We have a limited understanding about the hypothesized pathways between education and chronic HIV care. Literature on education and HIV treatment adherence relationships has largely been constrained to measuring ‘education’ only in terms of the quantity of schooling obtained (Chamberlin, 2022; Chamberlin et al., 2021; Heestermans et al., 2016). Population-level analyses in the region have given minimal attention to the purported outcomes of formal education—including skills such as reading, writing, and arithmetic, or more lucrative employment—in relation to chronic care management. We are aware of only one study in The Gambia in western Africa that directly measured—and demonstrated a positive association between—literacy and viral load suppression (Hegazi et al., 2010). As such, it is unclear the extent to which different facets of ‘education’, beyond attainment, may influence HIV chronic care management in the region.

Intriguingly, we might expect differences in the relationship between educational attainment and chronic care management for men versus women, and older versus younger populations—giving us insight into how education influences chronic care. Men have greater access to formal employment opportunities when compared to similarly educated women; and older populations who were able to attend school received higher quality education and potentially greater skill sets than similarly educated younger populations (Chamberlin, 2022; Desmond et al., 2024; Pritchett, 2013). As such, it is plausible that more educated men’s greater income would better equip them to access HIV care, making education a stronger predictor of men’s HIV treatment adherence than for women. It is also possible that the greater skills among more educated older adults would give them an HIV treatment adherence advantage compared to their less educated counterparts; and that such an education disparity might not persist for younger adults who received fewer skills due to lower quality schooling. Yet, when assessed at the population level in southern and eastern Africa, the lack of a statistical association between formal education and viral load suppression persisted for both men and women, and populations of all ages (Chamberlin, 2022). This further suggests that the education-related skills and resources needed to potentially support chronic HIV care management may also operate in unexpected ways in the region.

It is possible that the skills and resources commonly linked to educational attainment, such as literacy and income, simply have no bearing on managing chronic HIV care in this setting. More likely, we suggest that education-related skills and resources may be relevant for chronic HIV care management, but that contextual realities and complex processes involved in on-going care and treatment may attenuate the relationship between formal schooling and chronic HIV care management. In this study we aim to gain deeper insight about what drives the unexpected lack of statistical associations between educational attainment and HIV treatment adherence. We use in-depth interviews from Malawi to learn how HIV clients experience and use education-related skills and resources in their daily lives to manage their HIV care and treatment. To better understand how these skills and resources fit (or do not fit) in the pathway between formal educational attainment and chronic HIV care management, we further compare narratives across respondents with different education levels.

### 1.1. Hypothesizing theoretical processes linking education and chronic HIV care management

**Fig. 1.**
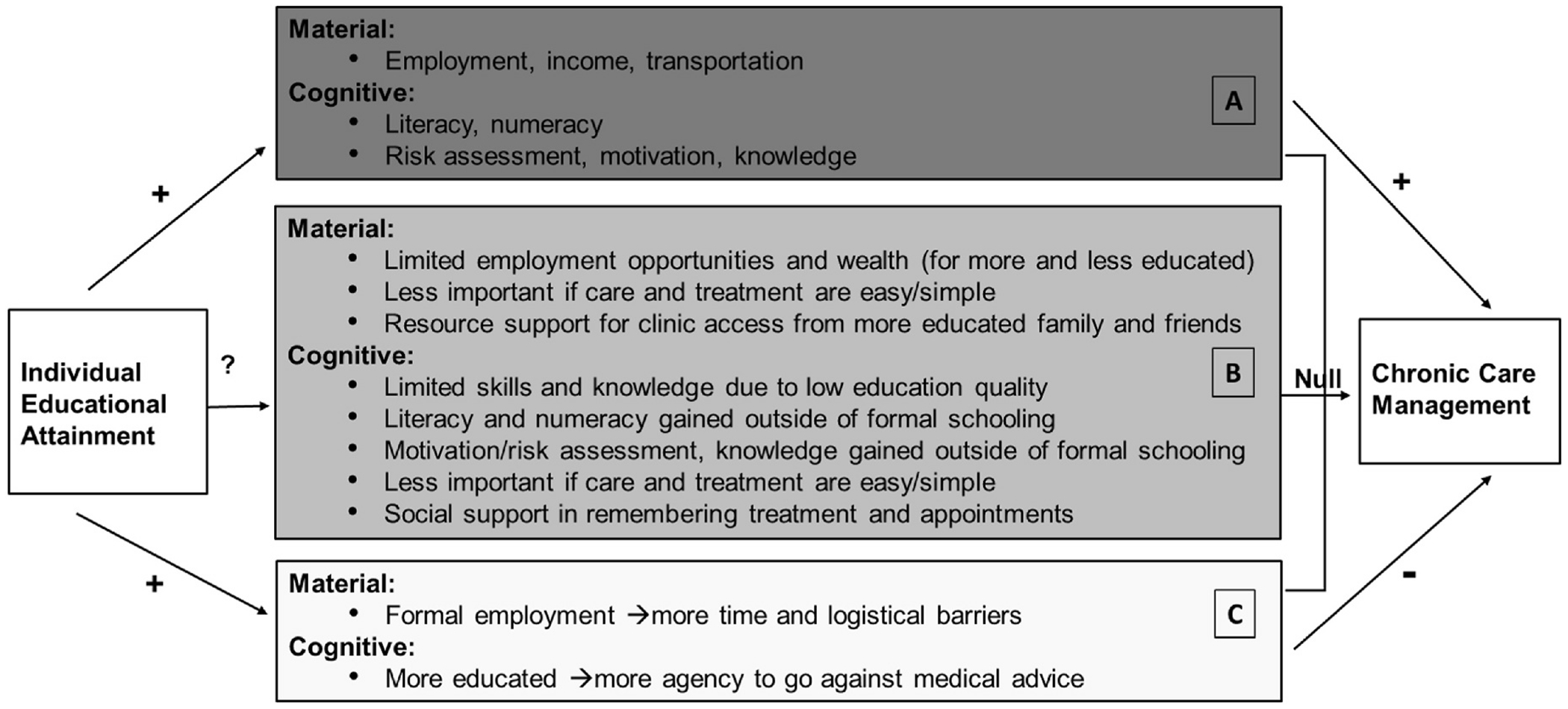
Summary of theoretical mechanisms. A. Dark grey box: mechanisms suggesting a positive association. B. Medium grey box: mechanisms suggesting a null association. C. Light grey box: mechanisms suggesting a negative association.

This research is responsive to frequent calls to examine the mechanisms linking education and health (Freese & Lutfey, 2011; Zajacova & Lawrence, 2018). In Fig. 1, we outline hypothesized theoretical mechanisms that may influence the link (or lack thereof) between formal education and chronic HIV care management, based on our review of the extant education-health literature. We have summarized those theoretical mechanisms using dark grey (positive), medium grey (null), and light grey (negative) boxes to demonstrate the possible direction of the association between education and chronic HIV care management when considered separately and jointly. Broadly, we categorize the resources conferred by education as ‘material’ or ‘cognitive’. Material resources are those externally derived, fiscal and tangible tools that can be used to access health services. Cognitive resources are internally held skills, knowledge, and motivation used to make health decisions and manage chronic care tasks. Considering these two sets of resources separately allows us to more precisely discuss the mechanisms that may link individual education, independent of other socio-economic factors, to chronic HIV care management.

The top, dark grey box (A) outlines the most commonly hypothesized pathways between education and health, resulting in positive associations between education and improved chronic care management. Education is globally theorized as a key means for gaining more lucrative employment, which may in turn facilitate access to health care (Cutler & Lleras-Muney, 2006). In Malawi, HIV care clients must obtain medication refills from the clinic on a monthly or quarterly basis, often requiring travel of more than an hour one-way (Malawi Ministriy of Health and Population, 2018). Most families in Malawi do not own a vehicle and rely on public transportation, bicycles, or walking—significant investments of money and time (Palk et al., 2020; Yeatman et al., 2018). Thus, from a logistical perspective, material resources, in the form of transportation and time, are critical for accessing the clinic.

Affording access to the clinic, however, is only one part of the work of chronic care management. Managing a chronic condition like HIV also requires strategies to remember and take daily medication and to return to the clinic for medication refills and routine check-ups. In many parts of southern and eastern Africa, tools such as a hard copy ‘health passport’ are used to record appointment dates and pill numbers—tools that require reading and writing skills. In a setting like Malawi, where 30 % of the adult population lack even basic reading skills, the more educated who can read and write may have a particular advantage in adhering to their chronic care (World Bank, 2024). Indeed, higher maternal literacy is linked to greater uptake of preventative health care for women and children in the region (LeVine et al., 2011; Smith-Greenaway, 2013; Hegazi et al., 2010); and, in Malawi, greater individual literacy is associated with better self-reported health (Smith-Greenaway, 2015). Other cognitive factors such as numeracy to count pills, accurate risk assessment regarding missed pills or appointments, and greater self-efficacy to navigate the health system are important facilitators for effective HIV care and treatment and are more prevalent amongst those with more education (Lipkus & Peters, 2009; Pinheiro et al., 2002).

These theoretical mechanisms (A) are premised on three key assumptions that: 1) people attending formal schooling actually gain the types of skills and resources that will help them manage chronic care from their schooling; 2) people do not derive skills and resources to support their health through other avenues, outside of their formal school attendance; and 3) people with more education will be more likely to share the same health objectives as those prescribed by health institutions. In reality, there may be myriad ways in which education systems, economic systems, health systems, and individual lives and priorities do not align with these assumptions.

In the medium (B) and light grey (C) boxes, we outline how these assumptions may not hold in the context of southern and eastern Africa. As noted in the medium grey box (B), there are three reasons to believe that material resources may not be a key mechanism by which education facilitates chronic care management. In fact, existing statistical evidence in the region, including Malawi, shows inconsistent relationships between HIV treatment adherence and employment, income, or wealth (Chamberlin, 2022; Heestermans et al., 2016). First, there are limited consistent employment opportunities, particularly in rural areas, and many of the income-earning opportunities that do exist are seasonal and do not require schooling credentials (Kaler, 2016; McCullough, 2015). Further, a majority of people who enter the schooling system do not complete secondary and tertiary education, which means they do not receive the credentials that are valued in the employment sector (World Bank, 2024; Kaler, 2016). As such, even those with relatively greater schooling may not achieve markedly greater levels of wealth and income.

Second, HIV care and treatment in the region is free for all HIV-positive adults and at the time of this study were available in most local health clinics (UNAIDS). In many other settings in the world, it is asserted that more education leads to greater access to employment-sponsored health insurance and/or money to pay for health services (Zimmerman, Woolf, & Haley, 2015; Moonesinghe et al., 2013). Given the context of free HIV care and treatment during this study, material resources for paying for health services are contextually less relevant for achieving HIV treatment adherence.

Third, individuals across southern and eastern Africa, including Malawi, rely on family and friends to access transportation and other resources to attend their HIV care appointments (Chamberlin et al., 2021; Ware et al., 2009). Moreover, living among more educated communities can have a positive benefit for a variety of health outcomes for the more and less educated alike (Benefo, 2006; Benjamin-Chung et al., 2017; Kravdal, 2002; Stephenson et al., 2007). This suggests that it may not be the individual’s education, but rather the financial and material resources available within an HIV client’s social network or community that facilitate their HIV care engagement. For these reasons, part of the commonly accepted theoretical rationale—that education would facilitate chronic HIV care through an individual’s greater material resources—may simply not hold in sub-Saharan Africa.

In terms of cognitive resources, those who do attend school in the region are often not achieving basic proficiency in reading and math (UNESCO, 2015). These gaps in skill attainment fuel growing concern among policy makers that, while access to schooling has increased, the quality of that schooling has not improved and has even diminished in some cases (Kaler, 2016; Pritchett, 2013). This is further demonstrated by Smith-Greenaway’s (2015) study in Malawi showing that literacy skills remained low even among those with eight-plus years of schooling. Moreover, chronic absenteeism, nutritional deficits, and limited time to devote to studying are common in the region, further limiting students’ abilities to learn in the classroom (Bloom, 2007). Thus, formal education may not fully provide the basic skills needed for chronic care management.

At the same time, those skills and knowledge may also be gained outside of the formal school system. Adult and non-formal literacy classes are growing more common, and can facilitate basic reading and writing skills development (Joshi et al., 2023). Further, numeracy skills can be acquired and practiced in non-educational settings like the market place (Banerjee et al., 2017). Similar to material resources, living amidst more literate communities and families, may positively influence that individuals health, especially if that individual is unable to read and write themselves (Basu et al., 2001; Smith-Greenaway, 2017). What is more, knowledge about the life saving power of HIV medication is now widespread in the region, where people have often witnessed the importance of treatment adherence amongst those in their social circles (Chamberlin et al., 2021; Gilbert & Walker, 2009; Helleringer & Kohler, 2005), suggesting that even those without education may be aware of the benefits and risks surrounding HIV treatment adherence. Thus, many of the cognitive skills that are needed to support chronic care management may be obtained outside of formal educational settings, potentially making formal educational attainment less critical to managing HIV care.

Relative to some other chronic conditions or previous HIV treatments, the task of managing HIV care may also be cognitively less burdensome. Today, HIV treatment typically only requires one pill per day, and periodic refills are fairly predictable. In addition, the HIV care system in southern and eastern Africa is robust thanks to international funding, and offers client’s numerous supports, including written and verbal appointment reminders and community support groups. Given these simpler treatment regimens and support systems, the cognitive skills that are needed to manage HIV care may also be simpler, requiring less education (Goldman & Lakdawalla, 2005). Moreover, many in the region report help from friends and family members for remembering to take pills and return to the clinic—a simple yet effective form of social support that exists for the more and less educated alike (Chamberlin et al., 2021; Ware et al., 2009). In summary, the factors listed in the medium grey box (B) indicate that the path between formal education and key cognitive and material mechanisms for chronic care management may be weaker in the Southern and Eastern African context.

Finally, in the light grey box (C), we outline ways in which education may inhibit chronic care engagement in some cases. Where education does provide access to more lucrative employment opportunities, that employment itself may make it difficult to take time off work in order to attend routine clinic appointments (Freese & Lutfey, 2011). This may be because work schedules are stricter or more demanding, or because people in more formal work settings may experience increased stigma-related fear regarding clinic attendance (Judd et al., 2022; Madiba & Kekana, 2013). Alternatively, those with more education may have greater self-efficacy to go against medical advice or create their own adaptations to treatment regimens. Examples of this in other settings include alternate vaccination schedules, or devising justifications for splitting treatment dosages (Reich, 2020; Wei et al., 2009).

The separate or combined influence of countervailing mechanisms, as represented by the medium (B) and light grey (C) boxes, may attenuate the positive relationships (dark grey boxes) between education and chronic care management in sub-Saharan Africa. The impact of these countervailing mechanisms may depend on how pervasive they are in the population. If these factors exist but are not prevalent, they could detract from but not fully attenuate the overall positive impact of educational resources for chronic care management (Lutfey & Freese, 2005). To the extent that these, or other countervailing mechanisms, are prevalent they may help explain the lack of any population-level association between education and chronic care management.

In-depth interview data are particularly well suited for elucidating not only *which* mechanisms are at play, but *why* these mechanisms work the way they do in a specific context. Identifying a mechanism as ‘countervailing’ in the linkage between education and chronic care management requires an understanding of how that mechanism is obtained and then used in the process of managing HIV care and treatment. For example, simply identifying literacy as a mechanism does little to elucidate the possibility that literacy may be obtained outside of schooling and/or the relative importance of literacy compared to other skills for managing simpler HIV regimens. Our qualitative approach provides narratives about the dynamic processes by which people manage their HIV care, and opportunities for unexpected processes and mechanisms to emerge. Further, this qualitative approach allows respondents to describe their beliefs about the value of education-related skills and resources, and to clarify how they make sense of their use (or non-use) of such skills and resources within the context of their life circumstances.

### 1.2. Policy relevance

This research has implications for achieving key international policy goals around more equitable health and education systems. Chronic care management, unlike other preventative or acute care-seeking behaviors, routinely takes place outside of the clinic in the daily lives of clients. Throughout the region, public health practitioners increasingly focus on interventions to support client’s self-management of their chronic care (Aantjes et al., 2014). Self-management interventions are most effective when adapted to individual client’s skills, resources, and life circumstances (Swendeman et al., 2009)—factors commonly associated with educational attainment. Yet, consideration of clients’ education, literacy, and numeracy levels is less common in the discourse around support for HIV clients. This study provides insight into those self-management processes and how they can be adapted to client education levels.

At the same time, education scholars and policy makers in the region are increasingly examining the promise and relevance of formal schooling for improved well-being in adulthood. A key concern is whether schooling, in its current form, is contextually appropriate for increasing access to material resources and/or adequate for developing the skills needed in adulthood (Chudgar et al., 2019; Pritchett, 2013). The present analysis of the links between education and chronic care management can inform the design of education and health policy and interventions that: a) harness the power of education for chronic care, b) are responsive to the chronic care needs of clients with different education levels, and c) leverage non-formal resources—such as community support groups—beyond schooling and clinic engagement.

### 1.3. Study setting

Malawi is a small land-locked country at the nexus of southern and eastern Africa. While the history, culture, and economy of the country are unique in many ways, Malawi shares several key characteristics—particularly its HIV care and schooling systems—with neighboring countries in the region. Since 1994, Universal Primary Education policies have guaranteed fee-free primary education at local government schools throughout Malawi and elsewhere in the region (Pritchett, 2013). While this has dramatically increased enrollment rates in formal schooling, it has nonetheless coincided with declines in school quality. At the same time, access to secondary and tertiary education has remained largely out of reach for many in the region—requiring school fees for tuition and travel to boarding schools in many cases (UNESCO, 2015). Since 2017, HIV services have been provided for free throughout the country and region at local government clinics, ensuring Universal HIV Treatment to anyone living with HIV (Nash et al., 2018). It should be noted that Malawi was the first country to implement both policies, making it a key focus for international policymakers, but other countries in the region were not far behind. The systems that ensure access to HIV care and basic education were well-established in Malawi at the time of this study and reflect similar systems in other countries in the region, providing a useful case for studying the pathways between formal education and HIV care among the adult population.

In other pertinent ways, Malawi’s socio-economic profile does diverge from its neighbors. First, despite its early success in implementing Universal Primary Education, the country has relatively more adults with less than 3 years of schooling, whereas many other countries in the region see a higher proportion of school dropout rates towards the end of primary or secondary schooling (World Bank, 2024). Second, Malawi has a relatively high-density rural population, placing particular strain on local health clinics and schools (World Bank, 2024). Third, the country is among the most impoverished in the region, with relatively limited economic opportunities even in urban settings compared to its neighbors. Many rural residents have limited employment opportunities beyond subsistence and agricultural work, and many family members are forced to travel to other areas to find employment. This type of economic precarity in rural life in Malawi mirrors that of rural areas in neighboring countries (Abay et al., 2021; Andersson, 2025). As such, the focus on rural Malawian communities in the present study is most applicable to other rural settings in neighboring countries.

## 2. Methods

Our qualitative findings are derived from a larger, mixed methods study conducted by *Partners in Hope*—a Malawian non-governmental organization and Presidential Emergency Plan for AIDS Relief (PEPFAR)/USAID implementing partner for HIV care and treatment. This larger study focused on understanding barriers and facilitators to HIV care under newly implemented ‘Test and Treat’ policies at HIV clinics receiving technical support from Partners in Hope, which included surveys alongside in-depth interviews with HIV clients and analyses of HIV care patterns from medical record systems. While the qualitative sampling frame and design have been described in detail in prior publications (Chamberlin et al., 2021), the analytic approach for the current study differs from other studies using this same data. During the summer and fall of 2017, we conducted in-depth interviews with clients at HIV clinics included in the larger study, with a specific aim of understanding the experiences of those clients with common patterns of intermittent interruptions in their HIV care across eight clinics in peri-urban and rural southern and central Malawi. The present study focuses on a sub-aim of understanding how education influenced respondent’s experiences with managing their HIV care. The primary data collection team included four trained Malawian research assistants (two men and two women), two Malawian field supervisors, and a lead investigator who designed and supervised the research.

### 2.1. Sampling

We purposively sampled 44 HIV clients with typical patterns of disengagement and re-engagement with HIV care from eight rural or semi-urban clinics in southern and central Malawi. Eligibility was assessed via medical chart reviews conducted by research assistants. Clients were invited for an interview if they were attending the ART clinic on a day when the study team was present, were ≥18 years of age, initiated ART for the first time in the previous 12-months, had been ≥14 days late for an ART appointment in the same 12-month period, returned to HIV care within 60 days after the missed appointment, and were non-pregnant/non-breastfeeding at the time of recruitment. This allowed us to capture the experiences of those who were currently engaged in managing their HIV care, but who had recently faced clear challenges to their HIV care adherence.

### 2.2. Data collection

Interview guides consisted of key themes for interviewers to cover regarding HIV care management, along with sample prompts. We developed interview guides in collaboration with community health workers and local stakeholders. We designed aspects of the interview guide to directly address the role of education in the process of HIV care management, including: a) the perceived importance of HIV care clients’ education, b) the importance of family or friends’ education levels in the provision of social support, and c) the importance of literacy skills. However, it should be noted that the majority of information regarding education-related skills and resources emerged, unprompted, during other portions of the interview, such as when exploring how clients remembered appointments or barriers to receiving timely refills.

All interviews were conducted by trained Malawian research assistants in Chichewa (the local language) in private spaces at local health facilities. Respondents provided informed consent for participating in the study, including the audio recording of interviews. We ensured all respondents understood that they had the right to refuse participation at any time. We provided refreshments for participants during the interview but did not offer any other incentive or compensation. If a respondent needed additional support, interviewers were trained to enlist the assistance of clinic staff.

### 2.3. Data analysis

Interviews were simultaneously transcribed and translated into English by trained Malawian transcriptionists. The study team reviewed each interview within two weeks of data collection to conduct timely quality checks and adjust interviewing procedures when needed. During these initial reviews we developed preliminary coding themes and wrote memos to identify over-arching narratives in respondents’ stories.

For the present study, we took an abductive analytic approach to make sense of the education-related variation in the processes and mechanisms that people use to manage their HIV care and treatment (Timmermans & Tavory, 2012). Abduction is neither inductive nor deductive and is centered on an iterative process of working between theoretical possibilities, such as those outlined in Fig. 1, and unexpected or paradoxical findings to develop new ideas about the social world.

After data collection was completed, two analysts conducted thematic coding using Atlas.ti. We reviewed the data for broad, a priori, domains of education-related skills and resources that are commonly theorized to link education and health—e.g., literacy, money, and knowledge. During the coding process, new themes and mechanisms emerged, such as motivation to adhere to treatment, numeracy, and skills and resources obtained through social support systems. We applied sub-codes for those instances when education skills and resources were described as facilitating HIV care and treatment, when the lack of such skills and resources were described as a hindrance for HIV care and treatment, and when those skills and resources were described as inconsequential for HIV care and treatment. Finally, we examined the processes involved in obtaining and implementing these education-related skills and resources by overlaying additional codes—for example, money obtained through work versus via asking a family member, or reading a health passport versus writing on a calendar. We then stratified analyses by respondent-reported education level to examine variation in the mechanisms and processes among those with different levels of education, which allowed us to understand the potential pathways between education and chronic care management more clearly.

Ethical approval for this qualitative research was provided by the Institutional Review Board at University of California Los Angeles (UCLA) and the National Health Science Review Committee (NHSRC) in Malawi.

## 3. Results and discussion

The 44 Malawian in-depth interview respondents included 21 men and 23 women whose educational distribution mirrored that of the general population (World Bank, 2024)—five had no schooling, 16 had one to three years of schooling which we define as low education, 20 had four to seven years which we define as average education, and three had eight or more years of formal schooling which we define as a high level of education. Nearly all respondents reported earning income through piecework (i.e., ad hoc, part-time activities) or farming activities. The majority were below the age of 40, in an on-going relationship, had more than 2 children, and traveled over an hour to reach their local health clinic. Among those in a relationship, most reported a partner who was also HIV-positive and taking ART, a potential source of social support.

### 3.1. Material resources

In this section, we explore the role of education in acquiring material resources—employment, money, and transportation—and the extent to which such material resources facilitated respondent’s HIV care management.

#### 3.1.1. Employment as a facilitator

Respondents across the educational spectrum reported limited employment opportunities and income precarity, with most earning income through piecework or farming—work that can be difficult to predict and which pays low wages. Consequently, they uniformly described difficulty finding transportation to the health clinic, and seeking ad hoc income-earning opportunities to gain additional transportation money to seek HIV care, as illustrated in these quotes:

“I sold maize so that I could find transportation to the clinic” (38-year-old partnered woman with low schooling)

“I grow maize. So, when I don’t have money for coming [to the clinic], I take the maize and sell it, and then get money for coming [to the clinic].” (44-year-old partnered man with average schooling)

“I sometimes miss the day to come [to the clinic] because I don’t have money. [when explaining why he missed a recent clinic visit] I was busy and I sold cassava, and after selling the cassava, I found the transportation money.” (21-year-old partnered man with a high level of schooling)

These findings suggest that financial resources are key to maintaining HIV care, but we did not find evidence that those with more education necessarily had greater access to greater income or money for transportation. This corresponds with evidence showing that type of employment and income levels are similar for those across the education spectrum, particularly in rural areas of sub-Saharan Africa (Al-Samarrai & Bennell, 2007; Fox & Gandhi, 2021). These respondents’ experiences provide important contextual nuance to the common premise that education will facilitate HIV care maintenance via increased access to employment opportunities in this setting.

#### 3.1.2. Employment as a barrier

At the same time, the unpredictable nature of agricultural and piecework often results in erratic schedules and unexpected travel demands, which can interfere with HIV care appointments, especially for men. There was no evidence that education buffered (i.e., protected) respondents from these additional work-related challenges to HIV care. The following quotes are from men representing varying education levels, who describe their work-related challenges to accessing HIV care:

“Some days we do some work. So, we miss the [appointment] date … [sometimes it’s] farming, sometimes it is burning charcoal …. because when we have burnt the charcoal we are supposed to check on it so it doesn’t get destroyed. But we end up missing the [clinic appointment] date.” (46-year-old partnered man with low schooling)

“I work as a builder … So that I can support my family. I find piece work in Mozambique. So, I was worried about waiting to get paid and when they paid us, I went back home, and it happened that I had missed my appointment dates [at the clinic].” (46-year-old partnered man with average schooling)

“I did not come because my business [selling second-hand clothes] is too involving. I walk a lot, so it happened that I went far and forgot to get the card [health passport], and [when] I got back I found that [too much] time has passed.” (41-year-old partnered man with a high level of schooling)

Although work can provide key resources for maintaining HIV care, the need to provide food and money for oneself and family may also undermine one’s HIV treatment adherence for the more and less educated alike. Further, those with average to high education were no better equipped to handle work-related challenges and competing priorities than their less educated peers. Finally, as noted in previous research with this sample, there were greater barriers for women (versus men) in the form of unpaid labor, and this was true across education levels (Chamberlin et al., 2021).

#### 3.1.3. Social support and the acquisition of material resources

Respondents across education levels also described borrowing modes of (e.g., cars or bikes) or money for transportation from friends or family members to help them access the HIV clinic:

“I say to myself that ‘tomorrow I am going to the hospital. So even though I have no transport money, I try to source it’ … It’s just my friends, we do help one another. If they are in need I also help them.” (29-year-old partnered man with average schooling)

“Sometimes my family tells me to take the bicycle, sometimes buying me bananas or squash or salad … Sometimes my sister escorts me here or stands on the line instead of me.” (34-year-old partnered man with average schooling)

“Yes, someone helped me with k500 [to get to the clinic]. I didn’t tell him that I need transport so that I can go and receive [HIV] drugs. I haven’t told him yet.” (21-year-old partnered man with a high level of schooling)

These patterns suggest that in situations of widespread economic precarity, social support will serve as a common ‘safety net’ for most community members, independent of their schooling. This form of social capital was also documented in Tanzania, Uganda, and Nigeria as a key mechanism by which HIV clients overcome barriers to HIV care (Ware et al., 2009). Indeed, informal social safety nets are a central feature in the informal economic structure of communities throughout the region (Foster, 2007; Kpessa-Whyte, 2018). As such, individually held material resources may be a less relevant mechanism linking individual education with chronic HIV care management in this setting.

### 3.2. Cognitive resources

Here, we examine how respondents used cognitive resources—such as reading, writing, counting, logical reasoning skills, and internal motivation—in the process of maintaining their HIV care.

#### 3.2.1. Literacy skills

Across the education spectrum, respondents discussed the importance of reading and writing appointment information, using a variety of literacy-based tools, in order to remember their HIV care appointments. The most commonly used tool was the ‘health passport’– a small notebook provided by the health clinic that clients used to keep track of key health information, including appointment dates. Other common tools included calendars, phone reminders, and writing on walls. The following quotes from respondents with some schooling illustrate simple and effective ways these literacy-based tools can be used to help remember HIV care appointments among the literate:

“I just write on the wall. When I see it on the wall, I just know that it is this date [to go to the clinic].” (55-year-old partnered man with low schooling)

“I have a phone at home and I save a memo of the date in my phone, so before I check in my book [health passport] I start checking on my phone and compare with my book [health passport] …” (42-year-old unpartnered woman with average schooling)

“[The clinic staff] write the date in the health passport. They indicate that on this date, you have to go to the clinic. So, I check in the health passport.” (40-year-old partnered man with a high level of schooling)

#### 3.2.2. Shared literacy

Most respondents with no or lower formal education reported difficulty using their health passports or keeping track of appointment dates on their own. Respondents described directly asking friends or family who were able to read and write to help with remembering and recording the date of their HIV care appointments. Others who could not read or write also described less-direct stratagies such as memorizing their appointment date and then listening to a neighbor’s radio, borrowing a calendar, or going to church to learn the date.

“… I go to people who know how to write, so they read for me and tell me the date I should come here … I went to someone to read for me, who told me that I will come here next week on Friday.” (49-year-old partnered woman with no schooling)

“I don’t have a calendar. But I like listening to the radio so that I can remember the dates … because [my neighbor’s] house is attached to my house so it is possible to listen [to his radio] from here.” (40-year-old partnered man with low schooling)

“Sometimes you ask someone who is educated to read your appointment dates in the health passport and when you realize that it is today, you start off and go to the hospital.” (30-year-old partnered woman with low schooling)

In these interviews, those who possess reading and writing skills relied on them for remembering HIV care appointments—findings that resonate with the public health literature demonstrating the importance of literacy for health (Baker, 2006; LeVine et al., 2011). However, in contrast to other literature on health literacy for understanding complex health information, our findings highlight the importance of basic literacy skills that can be gained through basic primary or nonformal education. This suggests that even modest increases in literacy capabilities can have important implications for individual’s capacity manage their chronic care.

Such literacy skills need not be held by the individual HIV client alone, as those without such skills rely on the literacy skills or tools of others around them. Similar to these findings, ideas about ‘proximate’ vs. ‘isolated’ literacy suggest that the benefits of literacy extended beyond the individual (Basu et al., 2000; Maddox, 2008). Our research corresponds with the work of other international education scholars, who demonstrate a need for a greater focus on household and community literacy as a means for improving the health and well-being of all community members in hard-to-reach areas (Basu et al., 2008; Prins & Zholdoshalieva, 2025). In sum, people with varying education levels used written tools to remember their HIV care appointments, but they differed in the extent to which they were able to use those tools independently.

#### 3.2.3. Numeracy skills

Numeracy is the ability to count, calculate, interpret numeric data, and apply numeric information to logically solve problems. In comparison to literacy, numeracy has historically received much less attention in public health discourse (Lipkus & Peters, 2009), but is now a focus in the Sustainable Development Goals and is increasingly recognized as a separate life skill essential for achieving well-being (Oketch, 2018). In this study, basic counting and reasoning skills played a key role in helping clients to remember to refill their HIV medications, but the role of numeracy differed by respondents’ education and literacy levels.

For respondents with less literacy, in the absence of calendars or similar tools, HIV clients also needed to know how their appointment date (often read to them by others) related to the current date – i.e., how many days/weeks remained until they needed to go to the clinic. The following quotes from respondents with low education described their processes of acquiring information about the current date, and then counting pills and/or days to ensure they refilled their medication before running out:

“When my phone was working, I could see the date on the phone. I didn’t forget. I know that we are supposed to go to the hospital on a particular day … when I go to church, they announce the date for that particular day. So I count from that Sunday up to the day that you have an appointment and I realize that on such a date, I am supposed to go and pick up medications.” (38-year-old partnered woman with low schooling)

“You can have a radio close so you can easily know that this is the date but it is difficult for us since we don’t have a calendar and we don’t have a phone. So we just remember by counting the days, and you check with the health passport … I also count the pills in the bottle to say the medicine that I am taking, how many are remaining for the rest of the month? I try to count so that I should know to say on this date, I should go and collect some more pills.” (40-year-old partnered man with low schooling)

“There’s my in-law who goes to the bank—I ask them what date it is today. Or if students are going to school, I ask them what date it is today … I know that my next appointment is on the 20th or 22nd or 27th or 28th, so I count so that the following week I may go to the clinic.” (32-year-old unpartnered woman with average schooling)

Respondents with high levels of education also used numeric processes to maintain their HIV care, but to a lesser extent. These more educated respondents described quickly checking their pill bottle to make sure they were not running low on pills. But they did not report using pill counting and day counting to ascertain their appointment dates in the way that less educated people did. For example:

“Okay. In order to remember the date, firstly, we see how many pills we are remaining with. Secondly, we see the health passport for the next appointment date.” (36-year-old partnered man with a high level of schooling)

These results correspond with the findings of researchers in the U.S., where better HIV medication management was linked to greater numeracy skills (Waldrop-Valverde et al., 2010). Specifically, the use of numeracy skills among those with lower literacy corresponds with prior literature demonstrating that numeracy was an important factor in accounting for HIV treatment pills in lower literacy settings (i.e., Ethiopia) but not in higher literacy settings (i.e., Lesotho) (Hirsch-Moverman et al., 2017). These interviews shed light on the role of basic computational skills, but do not necessarily speak to other numeracy skills such as risk calculations and interpretation of more complex numeric health information (Lipkus & Peters, 2009). Enhancing and encouraging accurate computational skills for medication self-management may be key to supporting HIV treatment adherence in populations with limited literacy.

#### 3.2.4. Motivation and competing life priorities

Respondents across all education levels expressed a strong dedication to taking their HIV medications, understood that missing medication doses could be detrimental to their health, and demonstrated accurate basic knowledge about how antiretroviral medication (ARVs) suppress HIV in the body.

“Because ARVs are our life. When we are taking ARVs we become healthy. I came to the clinic because I was afraid of my personal health to say if I don’t go to the hospital today then I will not be able to take medicine for a week. So if I don’t take medicine for a week then the virus will multiply. So the virus will work according to its best ability but I have to go to the hospital.” (34-year-old unpartnered woman with no schooling)

“I thought that if I just stay at home and not come to pick up medications, I may face some unexpected problems in the future, getting sick. So, my relatives were explaining that I may unexpectedly get sick unlike how a person normally gets sick and therefore it is better for you to go to the hospital now while your immunity is still high and pick up medications so that you should continue taking them. That’s when I sourced some money to come here at the hospital to pick up medications.” (27-year-old partnered man with average schooling)

“When you don’t take drugs the virus multiplies that’s why we are supposed take those ART drugs every day without skipping because when you skip, the drugs don’t work again.” (41-year-old partnered man with a high level of schooling)

These findings suggest that education level was not a differentiating factor in respondents’ knowledge about or motivation for adhering to HIV care and treatment. This universal dedication to ART adherence may seem somewhat counter-intuitive, as everyone in this sample population had a lapse in HIV care in the past year. However, as we demonstrate elsewhere (Chamberlin et al., 2021), nearly all respondents expressed deep regret for those lapses and made concerted efforts to return to HIV care as soon as possible.

Due to the pervasive nature of HIV in Malawi and the decade-long, widespread availability of ARVs, many people may recognize the severity of treatment lapses and HIV treatment adherence may be socially normative (Ware et al., 2009). This does not necessarily negate the importance of education for motivating chronic care adherence, but suggests that other contextual factors such as disease prevalence, severity, and awareness of treatment can influence education-chronic care management associations.

## 4. Summary and conclusions

**Fig. 2.**
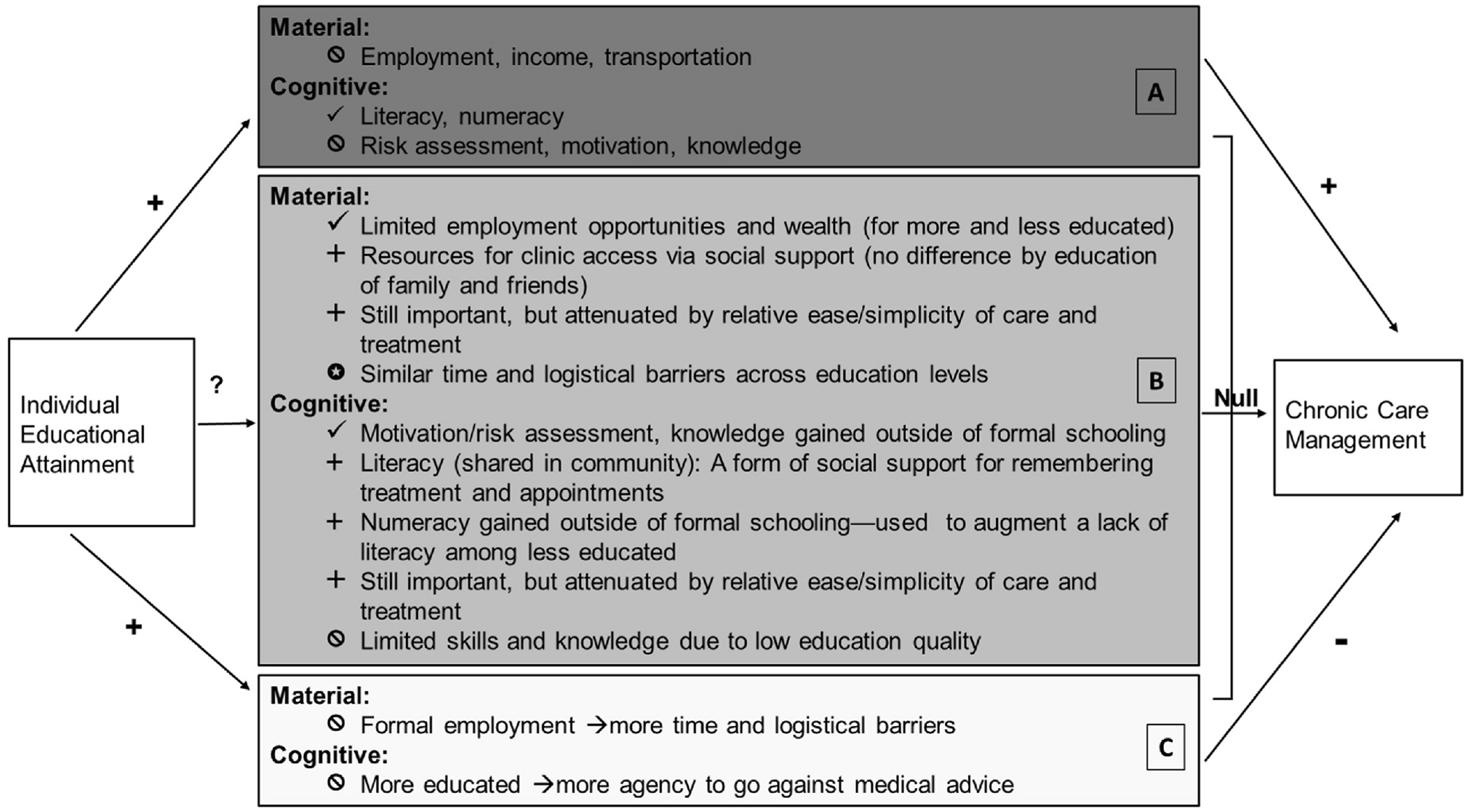
Summary: Theoretical and qualitative empirical relationships. D. Dark grey box: mechanisms suggesting a positive association. E. Medium grey box: mechanisms suggesting a null association. F. Light grey box: mechanisms suggesting a negative association. ✓ Theory-derived mechanism identified or confirmed in analyses. + Theory-derived mechanism identified in analyses, but operating in an unexpected way. ✪ New, unexpected mechanism identified in analyses. 🛇 Theory-derived mechanism not identified in analyses.

Our qualitative findings are consistent with other work that finds that education and chronic HIV care management are independent from one another (Chamberlin, 2022; Heestermans et al., 2016). We demonstrate the importance of cognitive and material resources for HIV care, as well as the notable countervailing mechanisms that attenuate such relationships. In Fig. 2, we summarize our empirical findings by adapting Fig. 1 from the Introduction—we highlight support for some of the mechanisms, while finding little evidence for other mechanisms originally outlined in Fig. 1. We also note some unexpected countervailing processes that newly emerged from these interviews. In short, material and cognitive resources appear to be central to maintaining HIV care, but the processes by which these resources are obtained and used potentially mitigates associations between formal schooling and HIV treatment adherence.

We offer three key insights related to material resources. First, in the context of limited economic opportunities, having more education may not differentiate the types of attainable employment, thus attenuating education’s effect on material resources through income. Second, in low-resource contexts, education may not be a distinguishing factor in who faces logistical obstacles to obtaining HIV care, or in who has the resources to overcome such logistical hurdles. Third, people across the education spectrum in resource-constrained settings tend to rely on their social networks for material resources. Many material resources needed to access HIV care are acquired through informal work or social networks, rather than via individual education-related mechanisms, which reinforces the importance of social safety nets in resource constrained settings. We suggest that on average in a low-resource population, income, wealth, or better employment opportunities are unlikely to mediate the relationship between education and effective chronic care management.

Our qualitative findings further demonstrate that key cognitive resources such as literacy, numeracy, knowledge about HIV treatment, and motivation to adhere to treatment are essential for facilitating HIV care management. However, the way these resources were obtained and deployed notably differed between those with different education levels. First, basic literacy clearly differed by education level, suggesting that even amidst concerns about low-quality schooling, formal education may facilitate these basic skills. However, we identified a somewhat unexpected countervailing process—those who were illiterate relied on literate community members to help them remember HIV appointments, which may attenuate links between individual education and HIV care management. Second, numeracy was used to remember HIV care appointment dates by respondents across the education spectrum, making it less clear if basic numeracy was obtained through formal education and/or other informal learning opportunities. Unexpectedly, when people’s literacy skills were limited, respondents relied more heavily on their basic numeracy skills to calculate when they should return to the HIV clinic—further attenuating the importance of individual literacy as a mechanism. Third, nearly all respondents, regardless of education level, expressed and demonstrated strong knowledge about the importance of HIV treatment, which produced a robust motivation to remain adherent to their treatment. This finding, in particular, contrasts with the common discourse in the fields of international development and global health, which tends to explain individuals’ less health-promoting behaviors as a function of their lower education levels.

It is notable, however, that only the most basic literacy and numeracy skills seemed important for on-going efforts to manage HIV care, and everyone in this sample was able to access HIV care—even if sporadically. This supports the idea that the simpler nature of HIV care and treatment, and the international funding for HIV services in the region, may somewhat minimize the need for education-related skills. This sentiment is reflected by multiple respondents in this study, who when asked directly if education helps people manage their HIV care, shared responses similar to this one:”*You don’t need school. You just have to take the drugs and don’t stop.” (21-year-old partnered man, 3 years of school)*. For other, more complex chronic health regimens in the region, the same may not be true. For example, calculating and measuring insulin doses to manage diabetes would certainly require greater numeracy skills, and the material resources needed for cancer treatments outside of local health centers would be substantially higher. Further, because of the substantial support for HIV services, largely through PEPFAR, there are numerous interventions to help clinics provide quality HIV services, remind clients about appointments, and counsel clients on HIV treatment adherence. Such supports are not widely available for other chronic conditions, and may dissipate for HIV care in the face of global health funding cuts. Thus, educational disparities for other chronic conditions may be present, and may emerge for HIV care in the future.

### 4.1. Limitations

It is important to note that this abductive analysis is intended to offer plausible mechanisms rather than providing conclusive evidence. Readers should consider these plausible mechanisms in light of several key study limitations. First, our in-depth interviews do not include those HIV clients with more extreme outcomes—those who are no longer engaged in HIV care or those who never lapsed in their HIV care attendance. It is possible that there would be more noticeable differences in the process of HIV treatment adherence across education levels had they been included. Nevertheless, the sample population we included is the most appropriate for understanding *typical* processes of on-going HIV care and treatment. Second, our sample only included three respondents with greater than primary education. It is conceivable that interviews with more educated individuals may have revealed starker differences in access to resources across education levels. However, it is important to note that even when individuals gain greater education, employment opportunities remain limited in rural areas, constraining opportunities even for the most educated living in these communities (Swidler & Watkins, 2009). Third, because we relied on client reports of both educational attainment and their self-described counting and reading activities, rather than a formal assessment of skill levels or reviewing education documentation, there may be some recall bias or mis-categorization of the respondent’s education and skill levels. Fourth, the sample includes a rural population in one country and the findings may not directly apply to other countries in the region. For example, there may be differences in employment opportunities in more urban areas or in countries with different socioeconomic profiles from Malawi. That said, prior quantitative research across different sub-Saharan African countries, including Malawi—with different educational and employment distributions—shows remarkably consistent null associations between formal educational attainment and HIV treatment adherence, suggesting that similar mechanisms may be at play in other settings (Chamberlin, 2022). Despite these potential limitations, this study provides important considerations for future testing and exploration in other countries and regions.

### 4.2. Program and policy implications

This research has direct implications for chronic care self-management interventions in the region, which are most effective when they respond to a client’s unique skills, resources, and life circumstances (Genberg et al., 2019; Kafu et al., 2020). A concerted effort has been made to increase the accessibility of HIV treatment, to tailor HIV care for lower resource contexts, and to instate differentiated care models that provide different means for accessing medications. Nevertheless, we are unaware of specific HIV policies or interventions that aim to meet the needs of those with different educational backgrounds, skills, and resources. Our findings suggest that factors such as literacy and numeracy skills and economic resources should be considered alongside other life factors when counseling clients about their HIV care. Respondents in our study with low literacy skills specifically requested such support: *“The request for those of us who cannot read is that we should be given something so that we are able to remember the date.”(47 year old unpartnered woman with no schooling)*. As an example, numeracy strategies to support those with lower literacy could be integrated with treatment adherence counseling. We found numerous instances in our interviews where respondents shared conflicting advice about how to use counting to remember appointments—wait until pills are gone to return to the clinic, always go before the pills run out, save your ‘buffer’ pills just in case, and even getting confused about pill numbers after sharing them with family or friends. Thus, while numeracy skills and strategies seem simple or basic, coaching clients on how to use pill and day counting in a consistent and effective manner can have real consequences for treatment lapses among the less literate. Many other repsondents directly requested that they be given and taught to use calendars—a pragmatic, yet simple intervention.

In terms of education policy, our research aligns with the Sustainable Development Goals that focus on what people do, and do not do, with their education outside the classroom (United Nations, 2023). As policy makers grapple with the best way to improve education quality and relevance, our findings demonstrate that the value of basic literacy skills for health should not be forgotten. In the short term, continued investments in non-formal adult literacy and numeracy interventions may have important returns to chronic care management in the region.

Finally, respondents’ reliance upon social networks to obtain resources for HIV care management poses a conundrum for social policy makers in the region. On the one hand, these informal social safety nets are essential and mitigate potential education-related health disparities and should be acknowledged as an important resource. On the other hand, these informal systems likely mask education-related inequities in the region (Kpessa-Whyte, 2018), as those with less education are able to engage with HIV care through the support of their social networks. Further, individuals dependent on informal supports likely experience greater precarity in managing their health, especially for a stigmatized condition like HIV, as they are dependent on the generosity of others and risk disclosure of their HIV status. A more explicit recognition of these informal processes may provide greater clarity about how education and health systems can be improved to support chronic care management. The findings presented here do not negate the potential contribution of formal education for the management of chronic care—material and cognitive resource needs were present but often met by others. Moreover, there may be greater education-related resource needs for other chronic conditions that will be too great for social supports to cover. Education policy designed to improve the relevance of education for people’s adult lives, should also consider the capabilities that individuals need to manage their chronic care with less precarious reliance on informal social safety nets. Public health policies in turn should focus on health system improvements that reduce the financial—treatment is free but getting to the clinic is not—and literacy-related demands often met by informal social safety nets.

### 4.3. Conclusion

Frequently, global health theory and research suggest that more formal education will facilitate better health behaviors and outcomes. Yet, in the case of southern and eastern Africa, educational attainment does not consistently predict HIV treatment adherence. The qualitative findings presented here from Malawi provide theoretical insight into the mechanisms that may attenuate education-HIV treatment associations at the population level. We demonstrate that the benefits from education for HIV treatment adherence are contextually shaped by limited employment opportunities, social support for transportation and literacy, and the acquisition of numeracy skills outside of the classroom. These interviews show the importance of material resources and cognitive skills—such as income or literacy—for managing chronic HIV care, but clarify that such skills and resources may be acquired outside of formal education. In light of the 2025 funding changes reshaping the landscape of HIV service delivery, it remains essential to continue monitoring the relationship between education and HIV chronic care outcomes. HIV programs that have recently been characterized by supportive, highly accessible services may decrease due to funding cuts, potentially exacerbating the need for education-related skills and resources to enable clients to successfully manage their chronic HIV care. Amidst health care system reforms, chronic care interventions (including HIV) should carefully consider adapting low-cost support services to assist individuals with different education-related skills and resources.

## Data Availability

Given the depth of information contained in the interviews for this study, the data have not been made publicly available to protect the confidentiality of respondents. The interview data that support the findings of this study are available from the corresponding author upon reasonable request.

## Code availability

The codebook, analytic memos, and the analytic codes attached to interview quotations in Atlas.ti are available from the corresponding author upon reasonable request.
